# Asymmetric relationships between proteins shape genome evolution

**DOI:** 10.1186/gb-2009-10-2-r19

**Published:** 2009-02-12

**Authors:** Richard A Notebaart, Philip R Kensche, Martijn A Huynen, Bas E Dutilh

**Affiliations:** 1 Center for Molecular and Biomolecular Informatics, Nijmegen Center for Molecular Life Sciences, Radboud University Nijmegen Medical Center, Geert Grooteplein 26-28, 6525 GA, Nijmegen, The Netherlands

## Abstract

**Background:**

The relationships between proteins are often asymmetric: one protein (A) depends for its function on another protein (B), but the second protein does not depend on the first. In metabolic networks there are multiple pathways that converge into one central pathway. The enzymes in the converging pathways depend on the enzymes in the central pathway, but the enzymes in the latter do not depend on any specific enzyme in the converging pathways. Asymmetric relations are analogous to the “if->then” logical relation where A implies B, but B does not imply A (A->B).

**Results:**

We show that the majority of relationships between enzymes in metabolic flux models of metabolism in *Escherichia coli *and *Saccharomyces cerevisiae *are asymmetric. We show furthermore that these asymmetric relationships are reflected in the expression of the genes encoding those enzymes, the effect of gene knockouts and the evolution of genomes. From the asymmetric relative dependency, one would expect that the gene that is relatively independent (B) can occur without the other dependent gene (A), but not the reverse. Indeed, when only one gene of an A->B pair is expressed, is essential, is present in a genome after an evolutionary gain or loss, it tends to be the independent gene (B). This bias is strongest for genes encoding proteins whose asymmetric relationship is evolutionarily conserved.

**Conclusions:**

The asymmetric relations between proteins that arise from the system properties of metabolic networks affect gene expression, the relative effect of gene knockouts and genome evolution in a predictable manner.

## Abstract 2

An investigation of metabolic networks in E. coli and S. cerevisiae reveals that asymmetric protein interactions affect gene expression, the relative effect of gene-knockouts and genome evolution.

## Background

**Figure 1:**
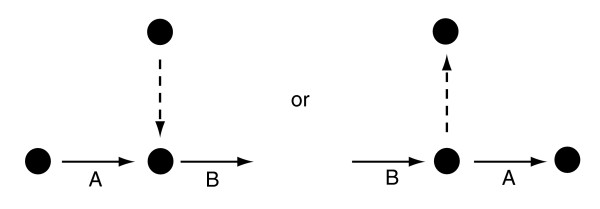
Simple examples of asymmetric relationship between reactions A and B (A→B). Nodes and arrows indicate metabolites and metabolic reactions, respectively. At steady-state the activity (that is, carrying a flux) of reaction A depends on the activity of B, but the activity of B is independent of the activity of A, because there is an alternative converging or diverging flux (dashed arrows).

Cellular processes can only be fully understood by considering how the functions of proteins depend upon each other. The relationship between two proteins can be symmetric - for example, when they mutually depend upon each other for their function within a protein complex. Proteins can also be asymmetrically related. This occurs when the function of one protein (A) depends on another protein (B), but the function of protein B does not depend on A: A→B. For example, in regulatory interactions, the function of the regulator depends on the presence of its target, but the target can often function without the regulator. Examples of asymmetrical relationships also exist in metabolism. For instance, multiple enzymes may produce the same substance (Figure 1), creating a situation in which the function of the proteins in the converging reaction fluxes (A) depends on the flux through B, but the function of B does not specifically depend on one of the converging fluxes. With the availability of accurate stoichiometric models of entire metabolic networks, it has become possible to infer symmetric and asymmetric coupling of reaction fluxes, not only at short metabolic distances, but throughout the complete network [1]. Asymmetrically coupled fluxes, when related to *in vivo *flux measures, do not exhibit a complete correlation (that is, symmetry) [2], and are much more frequent than the symmetric fully coupled fluxes (see below). Here we examine whether the asymmetric dependencies between proteins, as predicted from models of the complete metabolism of species at steady-state, are reflected in several genomic observables: which protein is expressed without the other, which is more essential than the other for survival or growth, which occurs in different genomes without the other and, finally, which is gained or lost without the other in evolution. To address these questions, we combined the dependencies of all reaction pairs in the metabolic networks of *Escherichia coli *[3] and *Saccharomyces cerevisiae *[4] with genome scale data sets for gene expression [5], gene essentiality [6,7], growth defects [8], and phylogenetic distribution [9].

## Results and discussion

Most coupled reaction pairs have an asymmetric dependency (that is, directional coupling): 82% in *Saccharomyces cerevisiae *[4] and 67% in the metabolic network of *Escherichia coli *[3] (see Materials and methods). As these asymmetric relations are so abundant in metabolism, we asked whether this characteristic is also reflected in other system properties of the cell. Given an asymmetrically coupled reaction pair A→B where A depends on B, but B does not depend on A (Figure 1), we expect that if one of the two reactions is inactive, it is most likely reaction A. To test this, we compared the asymmetric reaction pairs in the metabolic networks of *E. coli *and *S. cerevisiae *with four main types of genome scale data in which genes can be 'present' or 'absent'.

**Figure 2:**
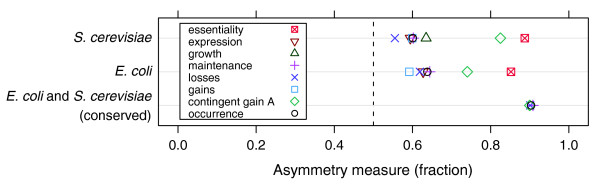
Asymmetrically linked reaction pairs (A→B) related to asymmetry in gene essentiality, growth defects, gene expression and genome evolution. The fraction (f_0/1 _= n_0/1_/(n_0/1 _+ n_1/0_)) where only B is essential in rich medium (essentiality) or has an effect on the growth across conditions (growth), where only B is expressed across conditions (expression), where only B is present across species (occurrence), where only B is present after gain, loss or maintenance over evolutionary lineages, and where A is contingently gained over evolutionary lineages (contingent gain A) is averaged over all reaction pairs (see Materials and methods). For conserved pairs there is no relevant result on gain, because too few (n = 2) events were found.

We first assessed the asymmetry in the lethality [6,7] and condition-specific growth defects [8] of gene knockouts. In an A→B situation, we expect that if only one of the two genes is essential or affects growth, this will be the B gene: in the absence of gene A, a flux may still flow through the reaction catalyzed by protein (gene) B, but without B, A cannot function. Indeed, we find that for 87% of the A→B pairs, in which one of the genes is essential, B is the essential gene (Figure 2; McNemar test; *S. cerevisiae*, n = 417; *E. coli*, n = 331; *p *< 10^-36^). The result for the condition-specific growth defects of non-essential A→B pairs is less pronounced, but still for 64% of the conditions, the loss of B causes a greater growth defect than the loss of gene A (Figure 2; two-sided Wilcoxon test; *S. cerevisiae*, n = 141; *p *< 2 × 10^-3^).

We also find a consistency of the asymmetric relations with gene expression patterns. Because gene A depends for its function on gene B, there should be few conditions where A is expressed without B, relative to situations where B is expressed without A. As expected, the B gene is expressed in 61% of the conditions where only one of two asymmetrically related genes is expressed (Figure 2; *S. cerevisiae*, n = 573; *E. coli*, n = 1,166; *p *< 10^-6^). In conclusion, these analyses show that asymmetric relations between metabolic enzymes are reflected in system properties of the specific organisms.

Next, we asked whether the asymmetric relations between enzymes are also reflected in evolution. Generally, functionally interacting proteins tend to co-occur across genomes [10,11]. This raises the question of whether the asymmetric relation of reactions is also reflected in the evolution of genomes. Although asymmetrically linked enzymes tend to co-occur [3], if only one of the two enzymes is absent from a genome, we expect this to be enzyme A: as A depends on the function of B, it will rarely be present in genomes where B is absent. To test this, we analyzed the phylogenetic distribution of all *E. coli *and *S. cerevisiae *A→B pairs across 373 species [9]. Indeed, gene A is the absent gene in 62% of the species where one of the two genes is absent (Figure 2; two-sided Wilcoxon test; *E. coli*, n = 1,225; *S. cerevisiae*, n = 2,242; *p *≈ 0). Besides asymmetry in the occurrence of genes in present day species, we also expect asymmetry in the gains and losses across evolutionary history. We inferred the occurrence of A and B in their ancestors by maximum parsimony [12]. In line with our expectations, gene A is more frequently lost (59%) in cases where a presence of both A and B in the ancestor was followed by a loss of either A or B (Figure 2; *E. coli*, n = 1,215; *S. cerevisiae*, n = 1,423; *p *< 10^-7^). Gene B is more often gained (60%) in cases where an absence of both A and B in the ancestor was followed by a gain of either A or B (*E. coli*, n = 605; *S. cerevisiae*, n = 1,449; *p *< 10^-6^). It is also expected that a gain of A depends on the presence of B (contingent evolution [13]). Indeed, a gain of gene A occurs more often when B is present (78%; *E. coli*, n = 824; *S. cerevisiae*, n = 1,472; *p *≈ 0) than when B is absent (see Materials and methods). Finally, there are also situations where a presence of only one gene in the ancestor is maintained along the evolutionary lineage (that is, neither of genes A or B were gained or lost). As expected, maintenance of A absent and B present was found more frequently than the reverse (62%; *E. coli*, n = 1,223; *S. cerevisiae*, n = 2,230; *p *≈ 0).

**Figure 3:**
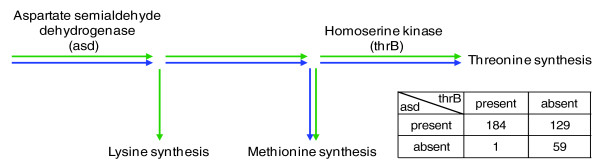
The asymmetric relationship between asd and thrB, two proteins conserved between *E. coli *(green) and *S. cerevisiae *(blue), is reflected in their asymmetric phylogenetic distributions. The activity of asd does not depend on thrB while the activity of thrB does depend on asd. Although in most cases both enzymes are present or absent together (243), thrB is more frequently absent while asd is present (129) than *vice versa *(1). The exception to the pattern comes from *Thermofilum pendens*, a species that has lost a large number of amino acid biosynthetic pathways, and imports most of its amino acids [14]. Note that a second asymmetric reaction pair between asd and the initial enzyme in the lysine synthesis pathway, present in *E. coli*, is not conserved in *S. cerevisiae*.

Although the various genomic and phylogenetic properties correlate significantly with the asymmetric relationships in the metabolic networks of *E. coli *or *S. cerevisiae*, exceptions remain where gene A is present while gene B is not. How can this be explained? For phylogenetic presence/absence patterns, one explanation for these irregularities is species-specific differences in metabolism. For example, the large scale replacement of amino-acid biosynthetic pathways by amino acid importers in *Thermofilum pendens *[14] has led to a situation where aspartate semialdehyde dehydrogenase (asd), one of the basal enzymes for amino-acid synthesis, is absent while homoserine kinase (thrB), which depends on asd, is still present (Figure 3). To examine such cases with unexpected phylogenetic occurrence systematically, we listed all asymmetrically dependent reaction pairs that lost gene A but not gene B in at least five monophyletic species (the expected pattern), and also lost gene B but not gene A in at least five monophyletic species (the unexpected pattern). Species with both genes present or both genes absent were allowed in both partitions (Additional data file 1). Some of these cases indeed reflect a change of metabolism, such as ubiquinone synthesis, which, in a species like *S. cerevisiae*, depends on the tryptophan biosynthesis pathway, while in *Homo sapiens *tryptophane is part of the diet and tryptophan biosynthesis has been lost but ubiquinone synthesis has been conserved. In most cases of unexpected loss, however, B has been replaced by a non-orthologous functional equivalent. Thus, the metabolic dependency of reaction A on B as identified in our reference metabolism may have remained intact, but the protein catalyzing B has changed. We also found cases of multiple functional specificities in orthologous group A, corresponding to a different substrate specificity of A in the species where B was lost, relative to the reference species *E. coli *or *S. cerevisiae *(Additional data file 1).

Even when genes and reactions are conserved across evolution, the nature of their relation can vary among species, as it depends on the overall functional and metabolic capabilities of the organism. Such variations could reduce the extent of asymmetry in the phylogenetic distribution. If this is the case, we expect to find a stronger correlation for genes with a conserved asymmetric dependency between the distantly related species *E. coli *and *S. cerevisiae *(see Figure 3 for an example). Indeed, we find a stronger correlation between the asymmetry in metabolism and the asymmetry in genomic occurrence across present day species and ancestral states if we consider reaction pairs with a conserved asymmetric relationship (n = 16) between the two studied networks (approximately 90%; Figure 2). Nevertheless, this set of conserved reactions has few exceptions to the predicted asymmetry which, like the exceptions above, can be explained by differences in the metabolism between species (Additional data file 2).

Having established that asymmetric dependencies derived from the metabolic networks are reflected in both species-specific system properties and evolution, we asked whether this correlation could simply be an effect of local network topology rather than the complete metabolism. We defined network distance between two reactions in the network as the minimal number of metabolites that separate them. For all the genomic properties studied, we find in most cases that the asymmetry is actually more pronounced at larger (non-trivial) network distances (d ≥ 4), with a fraction ranging from 56% to 99% (Additional data file 3). This shows that the asymmetric dependencies are not simply an effect of local network topology.

## Conclusion

We show here that the relationships between proteins that arise from their functional dependencies can have an important influence on other elements of the biological system. The analysis of relationships between genes has so far focused on symmetric relations, including correlated and anticorrelated phylogenetic distributions of genes, and on higher order logic [10,11,15,16]. Our findings underline the relevance of asymmetric binary relationships between proteins, such as those that can be inferred from metabolic networks, to explain the evolution and functioning of the system. We demonstrate that asymmetric flux relations between enzymes are more abundant than symmetric relations. Furthermore, we show that this asymmetry is reflected in gene expression, gene essentiality and the evolution of genomes, even for proteins at large metabolic distances. Our results suggest a potential to predict asymmetric functional relations between proteins on the basis of genomic data.

## Materials and methods

### Flux coupling analysis

Flux coupling [1] between reactions within the genome-scale metabolic networks of *E. coli *K12 (*i*JR904 GSM/GPR) [17] and *S. cerevisiae *iLL672 [18] was based on two recent studies [3,4]. Flux coupling relies on minimization and maximization of flux ratios (R_min _= lowest possible v_A_/v_B _ratio and R_max _= highest possible v_A_/v_B_) to determine the dependency between reaction A and B within the network (at steady-state [19]), given mass-balance constraints and flux capacity constraints (range of possible flux values; see also [1] for details).

In this study we mainly investigated the most abundant type of flux coupling, referred to as directional coupling (asymmetric dependency): the activity (flux) of one reaction (A) implies the activity of the other (B), but not necessarily the reverse (A→B, R_min _= 0 and R_max _= finite value). These reactions are coupled, but may not always operate together. In contrast, in fully coupled pairs (symmetric dependency) the activity of one reaction implies the activity of the other and *vice versa *(R_min _= R_max _= finite value). Calculations were done without assuming a constant biomass composition to avoid coupling of a large set of fluxes to the biomass reaction. All biomass components were allowed to be drained independently of one another (see [1,2] for details). Directional coupling between reactions was computed at a condition where all external nutrients were allowed for uptake and secretion (via capacity constraints on the exchange fluxes with environment) [3,4].

### Network distance

Network distances (d) were calculated by representing the network as a directed graph consisting of nodes (metabolites) and edges (reactions), and applying a shortest path algorithm. Distances correspond to the minimal number of nodes that separate any two reactions in the network. To increase the functional relevance of network distance, we removed the most highly connected nodes, including ATP, ADP, AMP, CO_2_, CoA, glutamate, H, NAD, NADP, NADH, NADPH, H_2_O, NH_3_, phosphate, and pyrophosphate [20].

We grouped directionally coupled pairs (A→B) into two network distance groups - close network distance (d < 4) and non-trivial distance (d ≤ 4) - to investigate whether the identified asymmetric relations are independent of network distance. Our conclusions are not affected by the exact distance cutoff between small and large network distance (Additional data file 3).

### Gene essentiality

Essentiality data for *S. cerevisiae *was obtained from the MIPS (Munich Information Center for Protein Sequences) database [7] (gene disruption table, 14-11-2005). Only essentiality information that referred to an original publication was retained, that is, database entries with a PubMed ID. If a gene was classified as both essential and non-essential by different sources, we assigned essentiality according to a majority rule and if no decision was possible, we marked the gene as ambiguous. For *E. coli*, we used the gene essentiality determined by Gerdes *et al*. [6]. We analyzed the essentiality on the level of reactions, using the gene-reaction associations as defined in each metabolic model. Reactions can be catalyzed by complexes of multiple enzymes (subunits linked by 'AND' in the model). Only if all subunits of an enzyme complex were essential did we consider the reaction essential. Conversely, only if all subunits were non-essential was the reaction considered non-essential. Otherwise, reactions were discarded. Reactions can also be catalyzed by iso-enzymes (linked by 'OR' in the model). If the individual iso-enzymes are classified as non-essential in single knockout experiments, it is still possible that the reaction is essential, because the loss of one iso-enzyme can be compensated by the other iso-enzymes. For this reason, we did not consider reactions with iso-enzymes. We summarized the combinations of essentiality and non-essentiality of all directionally coupled reactions in a 2 × 2 contingency table and tested for its symmetry by a McNemar test as implemented in R [21].

### Growth defects of gene knockouts

We used the condition-specific growth data of Hillenmeyer *et al*. [8] restricted to measurements at generation 5 of homozygous strains (12 conditions including dropouts of adenine, arginine, isoleucine, lysine, threonine, tryptophan, or tyrosine, as well as YP glycerol, minimal, sorbitol, synthetic complete media). We used the empirical *p*-values published by Hillenmeyer and co-workers [8] to derive binary profiles of significant (1) and insignificant (0) growth defects. To obtain unique *p*-values for every gene and condition, we calculated the geometric mean over batches, pools and scanners. A growth defect was considered significant if this average *p*-value was < 10^-3^. The mapping from gene to reaction level was done in the same way as for the essentiality data (see above). Subsequently, for each reaction pair A→B with a corresponding pair of growth effect profiles we calculated the fraction (f_0/1_) of conditions in which reaction A showed no growth effect while reaction B did (n_0/1_), relative to the total number of conditions in which only one of the reactions showed a growth effect (n_0/1 _+ n_1/0_). We tested the distribution of these fractions against the null-hypothesis that there is no bias, that is, no asymmetry (H_0_: f_0/1 _= 0.5), with the two-sided one-sample Wilcoxon test as implemented in R [21]. We averaged the calculated fractions over all pairs. For this and all other datasets, our results were qualitatively the same if we summarized the distribution as the mean or as the fraction of reaction pairs with a f_0/1 _> 0.5.

### Gene expression

The expression data were based on 13 studies with 327 conditions for *S. cerevisiae *and 12 studies with 420 conditions for *E. coli *(Additional data file 4). These data were obtained from the Gene Expression Omnibus (GEO) [5] at the National Center for Biotechnology Information (NCBI). Presence (expressed)/absence (not expressed) calls were made using the BioConductor affy package [22]. For each experimental condition, the presence/absence calls of individual genes were translated into 'presence/absence calls' of reactions based on the gene-reaction associations. Reactions that were catalyzed by multiple enzymes (iso-enzymes or subunits; see above) were considered present if at least one of the iso-enzymes or all subunits of enzyme complexes were present. For each reaction pair A→B with a corresponding pair of expression profiles, we calculated the fraction (f_0/1_) of conditions in which reaction A is absent while reaction B is present (n_0/1_) relative to the total number of conditions in which only one of the reactions is present (n_0/1 _+ n_1/0_). We tested the distribution of these fractions against the null-hypothesis that there is no bias - that is, no asymmetry (H_0_: f_0/1 _= 0.5) - with the two-sided one-sample Wilcoxon test as implemented in R [21].

### Reaction-level phylogenetic profiles and ancestral state reconstruction

We constructed phylogenetic profiles that denote the presence and absence of enzymes across 373 species according to the STRING 7.0 orthologous groups [9]. To explore the presence and absence of reactions across species, we mapped the enzyme orthology information to the reactions-level using the gene-reaction associations. In situations of iso-enzymes, we considered the reaction present in a species if at least one iso-enzyme was present. If a reaction was catalyzed by an enzyme that had multiple subunits, it was considered present in a species only if all these subunits were encoded in the genome. For each reaction pair A→B with a corresponding pair of 'reaction-level' phylogenetic profiles, we calculated the fraction (f_0/1_) of genomes in which reaction A is absent while reaction B is present (n_0/1_) relative to the total number of genomes in which exactly one of the reactions is present (n_0/1 _+ n_1/0_). We tested the distribution of these fractions against the null-hypothesis that there is no bias - that is, no asymmetry (H_0_: f_0/1 _= 0.5) - with the two-sided one-sample Wilcoxon test as implemented in R [21].

We inferred the most parsimonious ancestral presence/absence states of A and B using a phylogenetic tree of all 373 species included in this analysis (this tree contained some multifurcations to account for uncertainties [9]) and PAUP [12]. The tree was manually rooted at the trifurcation of eukaryotes, Eubacteria and Archaea. All results were based on a gain/loss cost ratio of 2/1 [23] and a delayed transition assumption ('DELTRAN'). Importantly, varying the parameters did not affect our conclusions.

We examined for each reaction pair A→B the following situations: type i, both reactions are absent in the ancestor and one is gained in the descendent; type ii, both reactions are present in the ancestor and one is lost in the descendent; type iii, the presence of exactly one of the reactions is maintained, that is, no change of state occurs. We calculated the fraction (f_0/1_) where B was gained (n_0/1_, type i) and where A was lost (n_0/1_, type ii) or maintained (n_0/1_, type iii) relative to the total number of instances of that type (that is, n_0/1 _+ n_1/0_). We tested the distribution of these fractions (over all AB pairs) against the null-hypothesis as mentioned above.

To analyze contingent gain of A, we determined for all gain events of A whether B was already present in the ancestor or not. The fraction of gains in presence of B (over all AB pairs) was tested against the null hypothesis that a gain of A is independent of the presence of B (that is, H_0_: f_gain _of A in presence of B = 0.5).

### Conserved directionally coupled reaction pairs

We considered a reaction to be conserved between *S. cerevisiae *and *E. coli *if it was catalyzed by orthologous enzymes. In the case of iso-enzymes we required that at least one orthologous enzyme was present in both organisms. For reactions catalyzed by enzyme complexes, we required that orthologs of all subunits were present in both organisms. The deviation of the asymmetry in gene gain, loss and maintenance was tested as discussed in the section 'Reaction-level phylogenetic profiles and ancestral state reconstruction'.

The absolute number of conserved directionally coupled pairs is limited (n = 16) because conservation of directional coupling required: both genes of a pair to be present in *S. cerevisiae *and *E. coli*; the type of coupling to be conserved; and the directionality (A→B) to be conserved.

## Authors' contributions

BD, RN, PK, MH conceived and designed the study. RN, BD and PK performed the analyses. RN, PK, MH and BD wrote the manuscript.

## Additional data files

The following additional data are available with the online version of this paper. Additional data file 1 is a table listing asymmetrically dependent reaction pairs A→B for which the independent gene B was lost while gene A was retained ('AB = 10') and vice versa ('AB = 01'), both in at least five species. Additional data file 2 is a figure that shows an exception to the predicted genomics occurrence of two enzymes. Additional data file 3 is a figure that shows asymmetrically linked reaction pairs (A→B) related to asymmetry in gene essentiality, growth defects, gene expression and phylogenetic distribution for which the pairs are categorized according to network distance cutoffs. Additional data file 4 contains two tables listing *Saccharomyces cerevisisae *[24-34] and *Escherichia coli *[35-44] expression datasets.

## Supplementary Material

Additional data file 1 Asymmetrically dependent reaction pairs A→B for which the independent gene B was lost while gene A was retained ('AB = 10') and *vice versa *('AB = 01'), both in at least five species. In this table, R is the smallest possible partition in the species tree (taken from STRING 7.0 [9]) that contained all 'AB = 10' species, and L is the remainder of the tree; we list only the cases where 'AB = 10' and 'AB = 01' were perfectly separable (neutral 'AB = 00' and 'AB = 11' species were not considered). Click here for file

Additional data file 2 The relation between fructose-bisphosphate aldolase (A) and the fructose bisphosphatase (B) is asymmetric in *E. coli *and *S. cerevisiae *because the gluconeogenesis contains an alternative flux that converges into fructose bisphosphatase. This asymmetry is, however, not reflected in evolution because fructose-bisphosphate aldolase occurs, as part of glycolysis, in a number of species in which gluconeogenesis and its specific enzyme fructose bisphosphatase are not present. This exception shows that the predicted asymmetry is not trivial, and depends on the conservation of the metabolism between species. Click here for file

Additional data file 3 The fraction (f_0/1 _= n_0/1_/(n_0/1 _+ n_1/0_)) where only B is essential in rich medium (essentiality) or has an effect on the growth across conditions (growth), where only B is expressed across conditions (expression), where only B is present across species (occurrence), where only B is present after gain, loss or maintenance over evolutionary lineages, and where A is contingently gained over evolutionary lineages (contingent gain A) is averaged over all reaction pairs (also see Materials and methods). Asterisk indicates *p *< 0.01. Click here for file

Additional data file 4 *Saccharomyces cerevisisae *[24-34] and *Escherichia coli *[35-44] expression datasets. Click here for file
