# Supplementary material for: Asymmetric relationships between proteins shape genome evolution
Source: Genome Biol. 2009 Feb 12;10(2):R19. doi: 10.1186/gb-2009-10-2-r19 (PMC2688278; doi:10.1186/gb-2009-10-2-r19)
Supplement: Additional data file 2 — The relation between fructose-bisphosphate aldolase (A) and the fructose bisphosphatase (B) is asymmetric in E. coli and S. cerevisiae because the gluconeogenesis contains an alternative flux that converges into fructose bisphosphatase. This asymmetry is, however, not reflected in evolution because fructose-bisphosphate aldolase occurs, as part of glycolysis, in a number of species in which gluconeogenesis and its specific enzyme fructose bisphosphatase are not present. This exception shows that the predicted asymmetry is not trivial, and depends on the conservation of the metabolism between species. [file gb-2009-10-2-r19-S2.pdf]

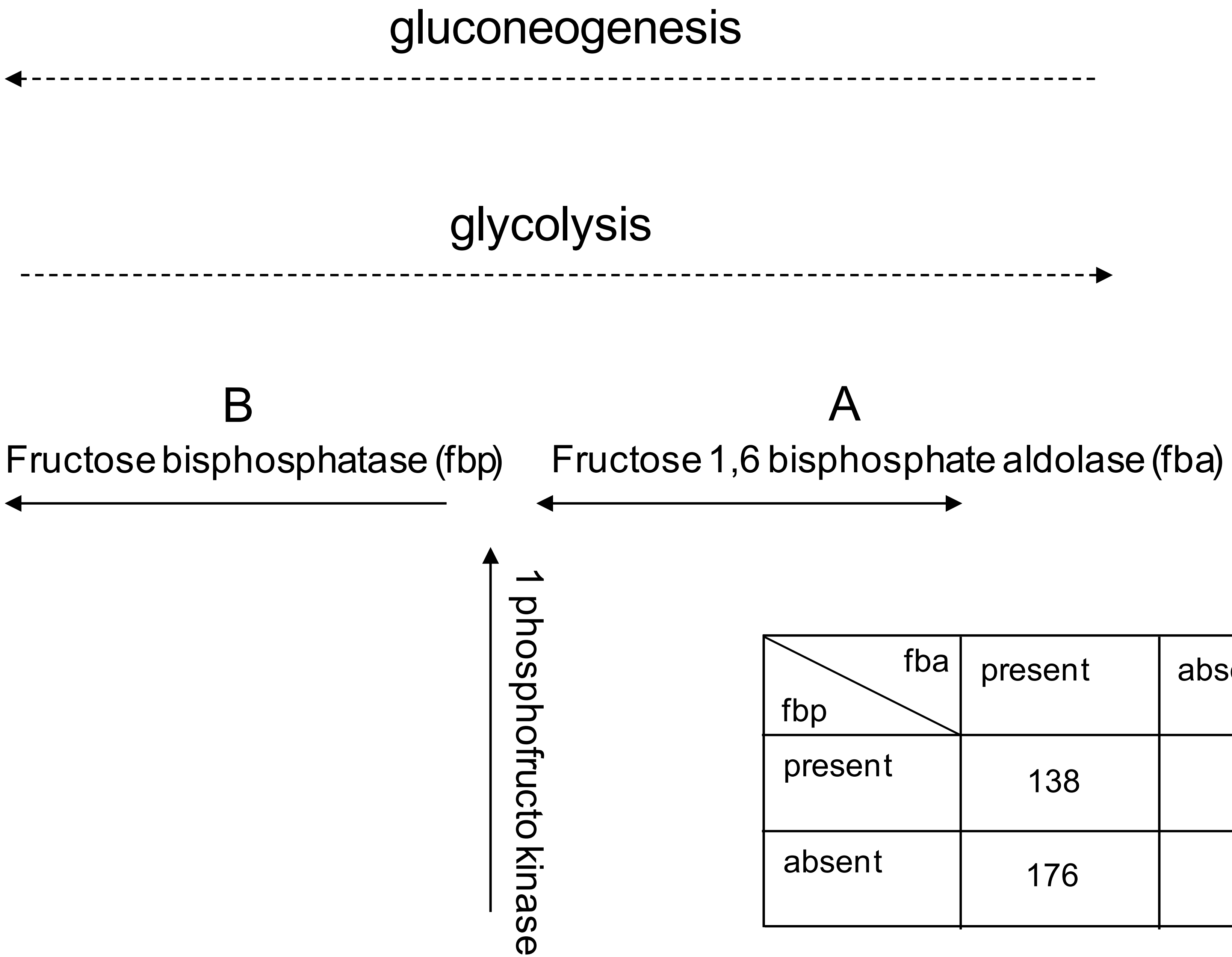

fbp \ fba	present	absent
present	138	23
absent	176	36
