# Supplementary figures and images for: Asymmetric relationships between proteins shape genome evolution
Source: Genome Biol. 2009 Feb 12;10(2):R19. doi: 10.1186/gb-2009-10-2-r19 (PMC2688278; doi:10.1186/gb-2009-10-2-r19)

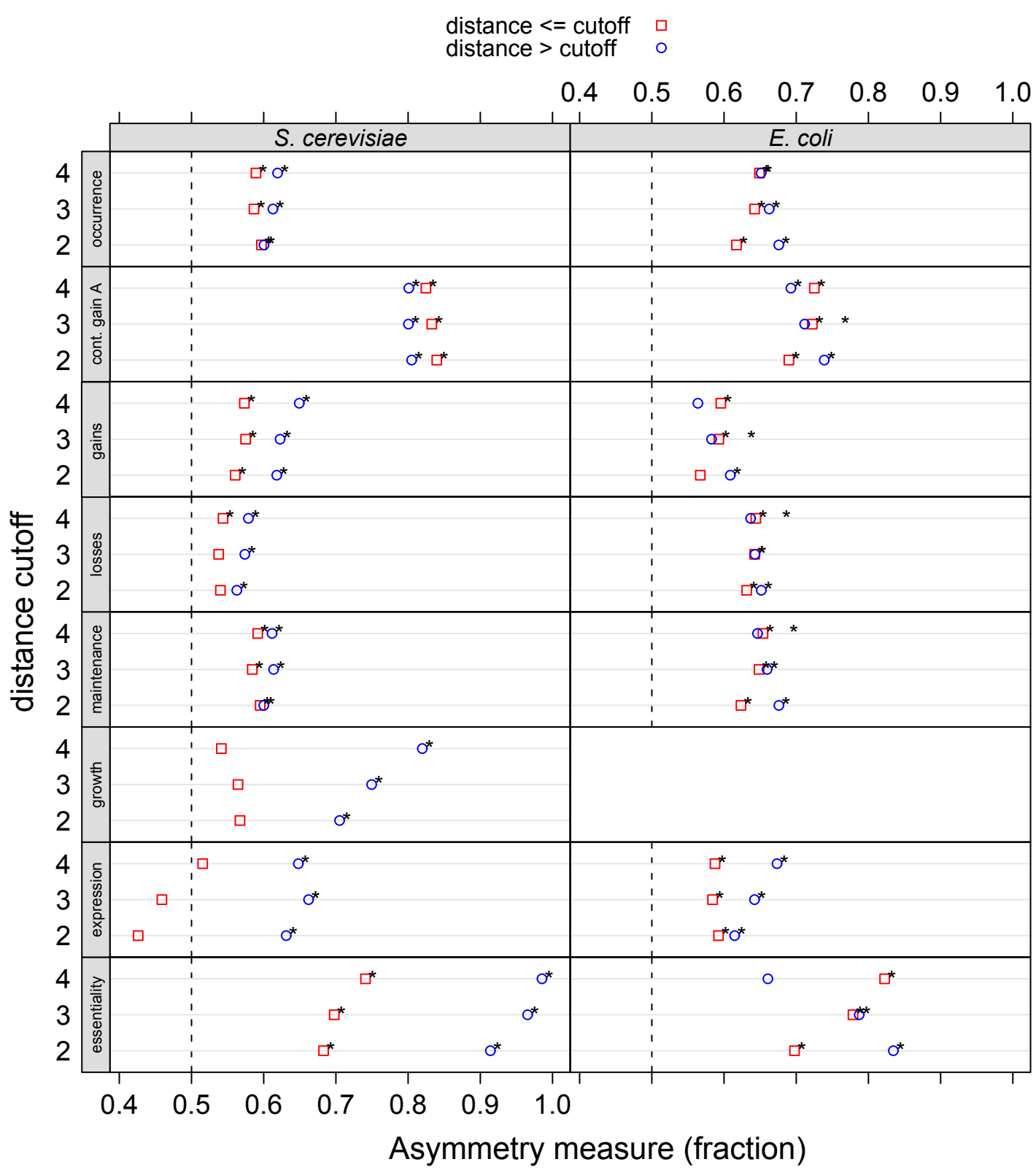

Supplement: Additional data file 3 — The fraction (f0/1 = n0/1/(n0/1 + n1/0)) where only B is essential in rich medium (essentiality) or has an effect on the growth across conditions (growth), where only B is expressed across conditions (expression), where only B is present across species (occurrence), where only B is present after gain, loss or maintenance over evolutionary lineages, and where A is contingently gained over evolutionary lineages (contingent gain A) is averaged over all reaction pairs (also see Materials and methods). Asterisk indicates p < 0.01. [file gb-2009-10-2-r19-S3.pdf]
