# Supplementary material for: Asymmetric relationships between proteins shape genome evolution
Source: Genome Biol. 2009 Feb 12;10(2):R19. doi: 10.1186/gb-2009-10-2-r19 (PMC2688278; doi:10.1186/gb-2009-10-2-r19)
Supplement: Additional data file 4 — Saccharomyces cerevisisae [24-34] and Escherichia coli [35-44] expression datasets. [file gb-2009-10-2-r19-S4.pdf]

Additional data file 4, table 1 and 2

Table 1. *Saccharomyces cerevisisae* expression datasets.

GEO series ID	Number of samples	Reference number as listed in main article
GSE6073	12	23
GSE1311, GSE1312, GSE1313	66	24
GSE1639	18	25
GSE1693	26	26
GSE1934	24	27
GSE1938	15	28
GSE1975	28	29
GSE2343	12	30
GSE3076	96	31
GSE3821	16	32
GSE4135	14	33

Table 2. *Escherichia coli* expression datasets.

GEO series ID	Number of samples	Reference number as listed in main article
GSE9814	6	34
GSE9755	4	35
GSE9582, GSE9587	8	36
GSE6925	2	37
GSE4562	7	38
GSE6425, GSE6426	54	39
GSE6836	266	40
GSE4556	15	41
GSE4511	15	42
GSE1121	43	43
